# Amidst spreading infectious diseases and climate change, US FDA should renew its focus on neglected tropical diseases

**DOI:** 10.1371/journal.pntd.0012005

**Published:** 2024-03-21

**Authors:** Mitchell Berger

**Affiliations:** US Federal Government, Rockville, Maryland, United States of America; University of Colorado - Anschutz Medical Campus, UNITED STATES

In 2015, the United States Food and Drug Administration (US FDA) and other Department of Health and Human Services agencies helped respond to a public health emergency concerning Zika Virus during which 5,600 cases of the virus were reported in the United States mainland and 37,000 cases in Puerto Rico and other US Territories [1]. In June 2023, the US Centers for Disease Control and Prevention alerted health providers to locally acquired malaria infections in Florida and Texas [2]. In October 2023, California (Pasadena) reported a case of dengue infection in a resident who apparently did not travel outside the United States [3]. In November 2023, an additional case report of cutaneous leishmaniasis in Texas seemed to confirm earlier suggestions that this disease had become endemic to the US [4]. These recent cases highlight one reason for US FDA to renew its past focus on developing policies and encouraging new diagnostics and treatments for neglected tropical diseases (NTDs).

Though primarily focused on the United States, US FDA considers itself to have an important global role. The agency, for example, supports a global program and international offices (see e.g., https://www.fda.gov/international-programs). Yet, even though, according to the World Health Organization’s 2023 Global Report on Neglected Tropical Diseases, nearly 1.65 billion people worldwide needed treatment for NTDs, and even amid such case reports as above, US FDA’s response to NTDs has for many years been fairly limited in scope and prominence [5].

In a well-written recent review in this journal, Mukherjee discusses US FDA programs relevant to NTDs and discusses existing or potential efforts such as additional incentive programs, enhancing the tropical disease Priority Review Voucher program, and use of authorities such as those covering orphan products that could spur additional NTD product development [6]. Yet, thus far as Mukherjee points out, most attention has focused on tuberculosis and malaria that are considered NTDs under US FDA’s definition but not those of the World Health Organization [5,6]. As explained by Mukherjee and other sources, Section 524 of the US Federal Food, Drug and Cosmetic Act and current US FDA guidance documents define neglected tropical diseases. The US FDA’s definition of NTDs includes malaria, tuberculosis, dengue/dengue haemorrhagic fever, leishmaniasis, and soil-transmitted helminthiasis (e.g., hookworm) as well as “[a]ny other infectious disease for which there is no significant market in developed nations and that disproportionately affects poor and marginalized populations” as designated by the Secretary of the US Department of Health and Human Services [6,7]. Thus, while Zika virus, tuberculosis, and malaria are considered NTDs by US FDA, they are not included on WHO’s list. Conversely, snakebite envenoming is considered an NTD by WHO but not US FDA.

Mukherjee points to several barriers to NTD product such as limited incentives and a difficult and lengthy process for bringing products to market [6]. However, another possible reason why NTD product development within the US has for been limited is lack of significant attention to NTDs by Congress and US FDA senior leadership. US FDA’s last major Report to Congress on NTDs, for example, dates to 2011 [8]. Its most pertinent guidance on the topic has not been updated since 2014 [9]. The last major US FDA hearing or workshop on NTDs was in 2010 [10]. While it is true that other US FDA programs may have overlap with and implications for NTD efforts, lacking in US FDA’s current or recent programs and policy development is an express focus on NTDs as has been the case in the past.

There are several reasons why US FDA’s taking a strong interest in NTDs or, seen from another perspective, resuming and updating its past activities would have benefits globally, for the American people, for drug, vaccine, medical device, and other manufacturers and even for US FDA and US FDA staff.

For one thing, climate change, development, and globalization are increasing the potential range of infectious diseases [11]. One recent review, for example, concluded that nearly 60% of 375 pathogens infecting humans may be exacerbated by climate change [11]. Focus on NTDs also would be consistent with US FDA’s efforts to enhance public health preparedness and response, including development of medical countermeasures [12].

US FDA could take several steps to bolster NTD research and product development. For example, US FDA could hold a new hearing or workshop on NTDs, updating its 2010 efforts. US FDA could update its guidances related to NTDs, including especially its 2014 Neglected Tropical Diseases of the Developing World: Developing Drugs for Treatment or Prevention. US FDA also could hold a new workshop or public hearing on this topic or issue a request for information to seek public input about ways it could enhance its NTD-related policies and programs.

US FDA also could form a working group comprised of US federal agencies that includes not only the National Institutes of Health but also, among others, representatives from the US Departments of Agriculture (animal health), Defense (research and efforts to protect military and dependents) and State (global diplomacy and health), US Patent and Trademark Office, US President’s Malaria Initiative, the Advanced Research Projects Agency for Health (*ARPA-H*) (Innovation), the US Agency for International Development (global programs), the Federal Emergency Management Agency (disaster response and planning), the US State Department’s US President’s Emergency Plan for AIDS Relief (PEPFAR) program, the Department of Health and Human Services’ Administration for Strategic Preparedness and Response and its Biomedical Advanced Research and Development Authority (emergency response and new product development), and the Centers for Disease Control and Prevention that includes its One Health program.

Ideally, a workgroup or collaborative, perhaps under the auspices of the Reagan-Udall Foundation for the [US] FDA (https://reaganudall.org/) or National Academies of Sciences, Engineering, and Medicine (https://www.nationalacademies.org/about) also could include representatives from the philanthropic and private sectors as well as medical and public health associations and state, local, tribal, and territorial public health agencies.

Even within US FDA, cross-agency partnerships and collaboration as NTDs span at least 4 US FDA Centers including those for Biologics Evaluation and Research, Devices and Radiological Health, Veterinary Medicine (i.e., Zoonoses), and Drug Evaluation and Research. CDER’s Medical Product Council could be one venue for holding such discussions as workgroups can include representatives from other Centers and Offices [13]. US FDA also can harmonize its NTD efforts with those of other national and international authorities such as the European Medicines Agency (EMA) and World Health Organization.

Recognizing that many existing drug, device, and biologic products could potentially be repurposed or repositioned for neglected tropical disease treatment, US FDA could build on and enhance its focus on drug, biologic, and medical device repurposing such as its December 2019 workshop with the National Institutes of Health and Reagan-Udall Foundation on repurposing off-patent drugs [14]. As some NTD treatments have developed over time without an ideal evidence base, US FDA, WHO, and others, including philanthropic and private sector partners, could review, where warranted, the evidence base for these treatments [15]. Through these and other steps, US FDA could significantly enhance its work on NTDs, providing critical leadership, coordination, and expertise at a time when such efforts are increasingly and greatly needed.

## Disclaimer

The author is a former consumer safety officer/senior policy analyst at U.S. FDA (CBER) and now works for another U.S. federal agency. The opinions expressed above are solely those of the author in his private capacity and should not be imputed to any public or private entity.

## References

[1] Government Accountability Office. Zika Supplemental Funding: Status of HHS Agencies’ Obligations, Disbursements, and the Activities Funded. Government Accountability. Office. 2018 May 14. PubMed PMID: [cited 2023 Nov 5]. Available from: https://www.gao.gov/products/gao-18-389.

[2] U.S. Centers for Disease Prevention and Control. Locally Acquired Malaria Cases Identified in the United States. Health Alert Network. 2023 [cited 2024 Feb 9]. Available from: https://emergency.cdc.gov/han/2023/han00494.asp.

[3] Schnirring L. California reports its first local dengue case Center for Infectious Disease Research & Policy (CIDRAP). 23 Oct. 2023 [cited 2023 Nov 4]. Available from: https://www.cidrap.umn.edu/dengue/california-reports-its-first-local-dengue-case.

[4] Barnhart M. A ‘tropical disease’ carried by sand flies is confirmed in a new country: the U.S. NPR Goats & Soda. 1 Nov 2023 [cited 2023 Nov 5]. Available from: https://www.npr.org/sections/goatsandsoda/2023/11/01/1209681147/leishmaniasis-sand-flies-tropical-disease-endemic-north-america-united-states.

[5] Global Report on Neglected Tropical Diseases 2023. World Health Organization. [cited 2023 Nov 5]. Available from: https://www.who.int/teams/control-of-neglected-tropical-diseases/global-report-on-neglected-tropical-diseases-2023.

[6] Mukherjee S. The United States Food and Drug Administration (FDA) regulatory response to combat neglected tropical diseases (NTDs): A review. PLoS Negl Trop Dis. 2023;17(1):e0011010. doi: 10.1371/journal.pntd.0011010 36634043 PMC9836280

[7] Food and Drug Administration. Tropical Disease Priority Review Voucher Program. 2020 July 15 [cited 4 Nov 2023]. Available from: https://www.fda.gov/about-fda/center-drug-evaluation-and-research-cder/tropical-disease-priority-review-voucher-program.

[8] Food and Drug Administration. FDA Report to Congress: Improving the Prevention, Diagnosis, and Treatment of Rare and Neglected Diseases. 2011 March [cited 2023 Nov 5]. Available from: https://www.fda.gov/about-fda/cdrh-reports/reports-congress https://www.fda.gov/about-fda/cdrh-reports/reports-congress.

[9] Guidance for Industry on Neglected Tropical Diseases of the Developing World: Developing Drugs for Treatment or Prevention; Availability. 7 July 2014 [cited 2023 Nov 5]. Available from: https://www.federalregister.gov/documents/2014/07/07/2014-15801/guidance-for-industry-on-neglected-tropical-diseases-of-the-developing-world-developing-drugs-for.

[10] Advancing the Development of Medical Products Used In the Prevention, Diagnosis, and Treatment of Neglected Tropical Diseases; Public Hearing. 20 July 2010 [cited 2023 Nov 5]. Available from: https://www.federalregister.gov/documents/2010/07/20/2010-17619/advancing-the-development-of-medical-products-used-in-the-prevention-diagnosis-and-treatment-of.

[11] Mora C, McKenzie T, Gaw I, Dean J, von Hammerstein H, Knudson T, et al. Over half of known human pathogenic diseases can be aggravated by climate change. Nat Clim Chang. 2022;12(90):869–875. doi: 10.1038/s41558-022-01426-1 35968032 PMC9362357

[12] Food and Drug Administration. Medical Countermeasures Initiative. [cited 2023 Nov 5]. Available from: https://www.fda.gov/emergency-preparedness-and-response/counterterrorism-and-emerging-threats/medical-countermeasures-initiative-mcmi.

[13] Food and Drug Administration. Center for Drug Evaluation and Research. Manual Of Policies and Procedures, CDER Medical Policy Council, MAPP# 4301.1 Rev. 3. 23 Feb 2021. [cited 2023 Nov 5]. Available from: https://www.fda.gov/about-fda/center-drug-evaluation-and-research-cder/cder-manual-policies-procedures-mapp.

[14] December 2019 Research & Regulatory Challenges Workshop, Therapeutic Repurposing, National Center for Advancing Translational Sciences, National Institutes of Health. 15 Jun 2022 [cited 2024 Feb 9]. Available from: https://sites.google.com/ncats.nih.gov/therapeutic-repurposing/home.

[15] Kappagoda S, Ioannidis JP. Neglected tropical diseases: survey and geometry of randomised evidence. BMJ. 2012;345:e6512. doi: 10.1136/bmj.e6512 23089149 PMC3478233

